# Online Gamma Radiation Monitoring Using Few-Mode Polymer CYTOP Fiber Bragg Gratings

**DOI:** 10.3390/s23010039

**Published:** 2022-12-21

**Authors:** Ivan Chapalo, Andrei Gusarov, Andreas Ioannou, Andreas Pospori, Karima Chah, Ying-Gang Nan, Kyriacos Kalli, Patrice Mégret

**Affiliations:** 1 Electromagnetism and Telecom Department, University of Mons, 7000 Mons, Belgium; 2 Belgian Nuclear Research Centre (SCK-CEN), 2400 Mol, Belgium; 3 Photonics and Optical Sensors Research Laboratory, Cyprus University of Technology, Limassol 3036, Cyprus

**Keywords:** polymer optical fiber, gamma radiation dosimetry, fiber Bragg gratings, gamma radiation, CYTOP, polymer perfluorinated fiber

## Abstract

We investigated the gamma radiation response of fiber Bragg gratings (FBGs) inscribed in a few-mode polymer optical fiber. The fiber had a graded-index CYTOP core of 20 µm and XYLEX overclad of 250 µm in diameter. Four FBGs were exposed to gamma radiation during four irradiation sessions at a 5.3 kGy/h dose rate. The FBGs showed a linear Bragg wavelength shift with the received dose with a mean sensitivity of −3.95 pm/kGy at 43 °C. The increased temperature provides a rise in the sensitivity: it reached −10.6 pm/kGy at 58 °C. After irradiation, the FBGs showed partial recovery, which increased with the received dose. Furthermore, the FBG’s reflection power decreased with the dose. This attenuation is mainly due to insertion losses caused by the radiation induced attenuation in the CYTOP fiber. Linear response to the received dose makes CYTOP FBGs attractive for gamma radiation dosimetry. However, temperature dependence of the sensitivity should be compensated in practical applications.

## 1. Introduction

Fiber optic sensing technologies allow remote and (or) distributed electromagnetically immune sensing of various physical quantities [1]. The influence of ionizing radiation on properties of optical fiber is an important field of research: optical fiber is often used in harsh environments under radiation exposure [2,3,4]. For the purpose of fiber communication and sensing under ionizing radiation, fiber transmission degradation is usually studied, and a proper composition of silica glass and dopants, resistive to radiation, is investigated [2,5,6]. In contrast, changes in the physical properties of optical fibers under irradiation are explored for fiber dosimetry. This field of research has been intensively developed in the last decades [5,7]. The interest in fiber optic dosimetry stems from the possibility of online and distributed sensing, remote interrogation, and small size of the sensitive elements. The main macroscopic reactions of silica fiber on ionizing radiation are radiation induced attenuation (RIA), radiation induced emission, and radiation induced refractive index change [5]. Generally, all of them can be used as radiation sensing mechanisms. In particular, dosimetry based on RIA is reported in various works [8,9]. Another sensing technology providing quasi-distributed dosimetry is fiber Bragg gratings (FBGs). Exposure to gamma radiation causes the Bragg wavelength (BW) shift, which can be mapped versus the received dose [10,11]. However, the sensitivity of silica FBGs decreases with received dose, tending the BW to saturate. Depending on the type of fiber, dopants, and inscription technique, the BW can saturate after receiving 20–100 kGy, providing a total BW shift up to 200 pm for 100-kGy dose [10]. A significantly stronger BW shift has been observed for several types of long period gratings (LPGs) [10,12,13,14]. In particular, chiral LPGs demonstrated a dip wavelength shift up to 10 nm at a 100 kGy dose and turn-around-point LPGs demonstrated a 80-nm shift at 65 kGy [13,14]. Such a stronger sensitivity can be an advantage, however, the nonlinearity of the response and the early saturation are still the main limitations of LPGs similarly to FBGs.

Recently, increased attention from researchers has also been focused on using a polymer fiber (POF) as a sensitive element for gamma- and X-ray dosimetry. Sensors based on the RIA effect in the visible range in polymetylmethacrylate (PMMA) POF demonstrated an advantage of stronger sensitivity over silica fibers [15,16,17,18]. Attenuation on unpaired electrons of free radicals created by a main chain scission under gamma radiation is the main mechanism of RIA in POF instead of attenuation on point defects in silica fiber [19]. Aside from the traditional use of fibers as sensitive elements, new sensing designs such as point sensors using the lab-on-fiber concept have recently been proposed. For example, a sensor based on metallo-dielectric resonator with a PMMA layer between two gold gratings located at the fiber end-face has been investigated as a proton radiation dosimeter of an ultra-high MGy dose scale [20].

Among other POFs, perfluorinated polymer optical fiber (PF-POF), which has a core/cladding structure based on CYTOP material and a polycarbonate overclad, is of strong interest for researchers and industry: it demonstrates radically low attenuation in telecom transparency windows of 850 and 1300 nm (down to 20 dB/km). It also has a higher bandwidth compared to other multimode fibers: 40 Gbit/s transmission rates over 100 m were successfully demonstrated [21,22]. Thanks to the femtosecond inscription method, various refractive index structures such as FBGs, long period gratings, and Fabry–Perot cavities were successfully created in PF-POF [23,24,25,26]. The effect of gamma and X-ray radiation on the PF-POF have also been investigated [19,27,28]. In particular, PF-POF demonstrated the RIA effect in UV–VIS range, significantly stronger than for PMMA [19]. Further development of PF-POF research allowed to propose distributed OTDR sensing based on RIA in PF-POF [29]. FBGs inscribed in PF-POF have also been investigated under radiation: blue shift of the FBG peaks as a result of gamma irradiation was observed in a standard 120-µm core graded-index PF-POF [30].

Recently, a custom few-mode graded-index PF-POF with a core diameter of 20 µm and an overclad diameter of 250 µm was developed. A smaller core diameter compared to the standard 50–120 µm PF-POFs limits the amount of mode groups possible to excite in the core by just a few (3–4 mode groups). This allows easier control of launching conditions, more stable mode power distribution during FBG operation, and, therefore, easier tracking of reflection peaks of the FBGs. The overclad diameter, similar to the one of the standard SMF-28 fiber, simplifies the process of fiber interconnection. Furthermore, different ratios between the diameters of the core/cladding structure and the overclad, which are made of different materials, could lead to differences in the sensing characteristics. For example, FBGs inscribed in the 20-µm fiber demonstrated a temperature sensitivity of 18 pm/°C, while for standard 50-µm FBGs, the values were found to be 28–38 pm/°C [24,31].

In this work, we investigate online the gamma radiation effect on FBGs inscribed in custom developed few-mode graded-index PF-POF with 20-µm CYTOP core and 250-µm XYLEX overclad. We demonstrate a blue shift of the BW under gamma radiation, as well as the linear response to received dose with average sensitivity of −3.95 pm/kGy at a dose rate of 5.3 kGy/h and 43 °C temperature. We analyzed both FBG irradiation and the BW recovery during a relaxation process. We also examined the impact of temperature on the BW response as well as the influence of PF-POF length on the reflection power.

## 2. Experimental Setup

**Figure 1:**
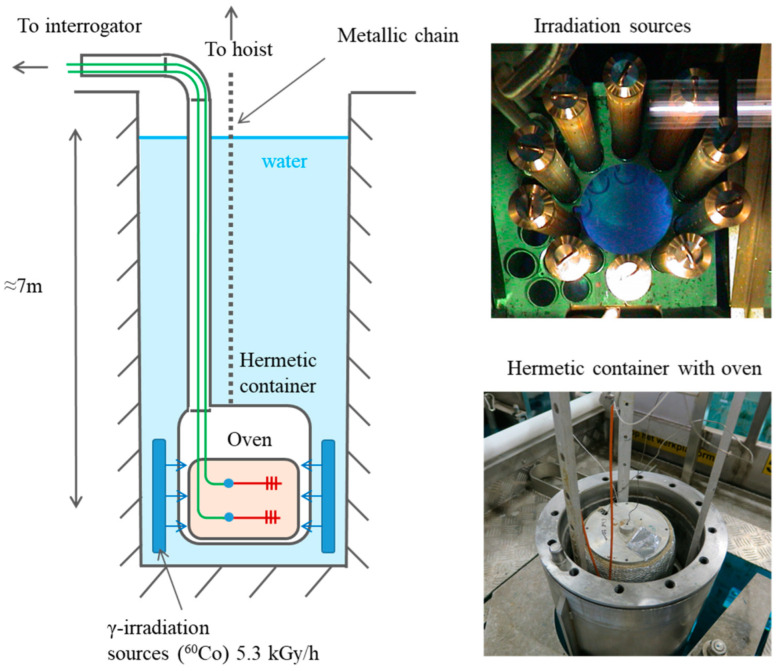
Schematic and photographs of the experimental setup.

The irradiation experiments with online measurements were conducted at a Brigitte irradiation facility (SCK-CEN, Mol, Belgium). The Brigitte consists of ^60^Co irradiation sources forming a cylindrical volume at a depth of seven meters in a water pool (Figure 1). Objects supposed to be irradiated are placed inside a stainless steel container, which can be sealed for underwater operation. The container is placed down to the irradiation zone for a specified time according to the required irradiation dose using an industrial manipulator (hoist). After irradiation, the container can be lifted up to a storage position above the water, or it can be lifted partly, to be stored under water at far enough distance from the irradiation sources. The last position is preferable for pre-irradiation stabilization and for the monitoring of post-irradiation effects thanks to more stable climatic conditions formed inside the container. A 9-m long tube with a diameter of 5 cm is hermetically connected to the container, so the optical and electrical cables can be passed from the equipment setup toward the investigated samples and thermocouples for online measurements. The irradiation sources provided a dose-rate of 5.3 kGy/h, which was measured before the experiments using the Harwell Red4034 PMMA dosimeters.

Gamma radiation causes heating of the irradiated material. We experimentally found that the temperature in the container during irradiation increased by 7 °C, which is quite significant for temperature dependent experiments. Therefore, we placed a small oven inside the container to perform experiments at controlled and stabilized temperature. The oven was driven by the Eurotherm 2408 controller. We first stabilized the temperature in the oven at 40 °C as the closest to the room condition on one hand, and to be far enough from the glass transition temperature of the POF (108 °C for CYTOP material) on the other hand. Stabilization at lower temperatures was not possible because the oven could only heat. However, during the first irradiation session, the temperature increased by 3 °C, up to 43 °C. After several tests, we stabilized the temperature at 42 °C, so its change during irradiation did not exceed 1 °C. Further increase in the temperature provided very slowly decreased the temperature rise during irradiation, so 42 °C was found to be the best compromise between temperature and its drift during irradiation.

The FBG samples were placed inside the oven and connected to 10-m standard SMF-28 patchcords passed through the tube. The opposite ends of the patchcords were connected to a commercial FBG interrogator FiberSensing FS2200 (1500–1600 nm spectral range, maximum acquisition rate is 1 measurement per second) for online monitoring of the reflection spectrum and storing the data. The interrogator had two input channels, therefore, we investigated two FBGs simultaneously during each irradiation session. The BW and the power of the reflection peak were defined using the original software of the interrogator.

**Figure 2:**
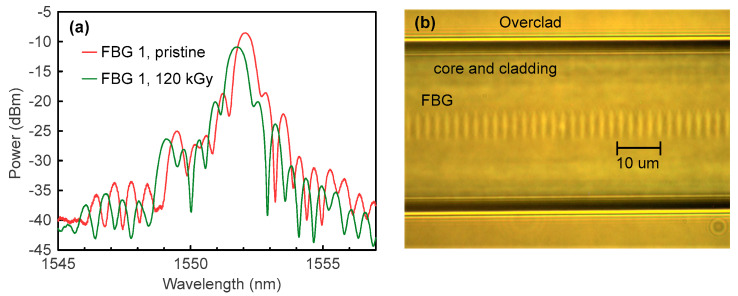
(**a**) Reflection spectrum of the FBG 1 before irradiation and after the second irradiation (120 kGy total dose); (**b**) transmission microscope image of a typical FBG.

In the experiments, we used the POF designed and produced by Chromis Technologies. It has a few-mode graded-index CYTOP^®^ core of 20-µm diameter and an effective refractive index of 1.34. A protective overclad of a 250-µm diameter was made of a XYLEX^®^ material, which is a blend of polycarbonate (PC) and an amorphous polyester. FBGs were inscribed by femtosecond (fs) pulses generated by a HighQ laser femtoREGEN source at λ = 517 nm (220 fs pulse duration and 1 kHz repetition rate). The BWs were chosen in the 1500–1600 nm window to be monitored by a commercial interrogator (in this range, the PF-POF had an ~200 dB/km attenuation that was acceptable for the lengths used in the experiments). We utilized a plane-by-plane direct inscription method to achieve better reflectivity and stability of the reflection spectrum. All FBGs had the same length of 1 mm, however, they could show different spectra due to slight differences in the launching conditions. The fiber samples with FBGs and the standard silica SMF pigtails were centered using two manual translation stages and connected using a UV-curing glue. A small core diameter of the fiber (comparing to standard GigaPOF-50SR CYTOP fiber with 50-µm core) and the flexibility of choosing desirable lateral dimensions of FBGs (thanks to a plane-by-plane technique) enabled us to achieve the reliable coupling of light into the fundamental mode, and as a result, a stable single-peak reflection spectrum even under slightly changing launching conditions. As an example, the reflection spectrum of the FBG 1 before irradiation and after two irradiation sessions (120 kGy total dose) is presented in Figure 2a; a transmission microscope image of a typical FBG is presented in Figure 2b. It is seen that the reflection spectrum after irradiation mainly contains the peak corresponding to the fundamental mode, similarly as before irradiation. This indicates that the UV-glue connection kept stable during irradiation and no unwanted offsets between silica and the CYTOP fibers occurred. The length of the POF between the FBGs and silica pigtails was 1–3 cm, except for FBG 2, which had a 1-m POF section. Prior to the experiments, all FBGs were annealed at 65 °C for 4 h to avoid fiber shrinkage and consequent permanent BW decrease during the experiments under increased temperature.

Experiments were designed for online measurement of the three main stages: under-water stabilization, irradiation, and post-irradiation relaxation. For stabilization, we placed the container with FBGs under water at a depth of 2–3 m and stored it until the BW was stabilized at a chosen temperature. For irradiation, the container was placed in the irradiation position as described above. After irradiation, the container was lifted up to the same underwater position for post-irradiation monitoring of the BW evolution. For the FBG replacement, we lifted the container out of the water and placed it on the storage position.

We performed three sessions of irradiation at 43 °C: 40 kGy, 80 kGy, 80 kGy, and one additional session of 80 kGy at an increased temperature of 58 °C (Table 1). Four FBGs were involved in the experiment. The FBG 1 was irradiated during all four sessions in order to investigate the cumulative effect of high total dose. In the first irradiation, FBGs 1 and 2 received a 40-kGy dose. Then, after relaxation at the underwater position, these FBGs were irradiated again for an additional dose of 80 kGy. The FBG 2 was then removed. FBG 3 was installed instead of FBG 2 and irradiated (together with the FBG 1) during irradiation 3 from a pristine state up to an 80 kGy dose. This was to investigate the response of the pristine FBG during receiving a higher dose (80 kGy) compared to irradiation session 1 (40 kGy). Finally, FBG 4 was installed instead of FBG 3 for irradiation 4 performed at the increased temperature of 58 °C.

## 3. Results

**Figure 3:**
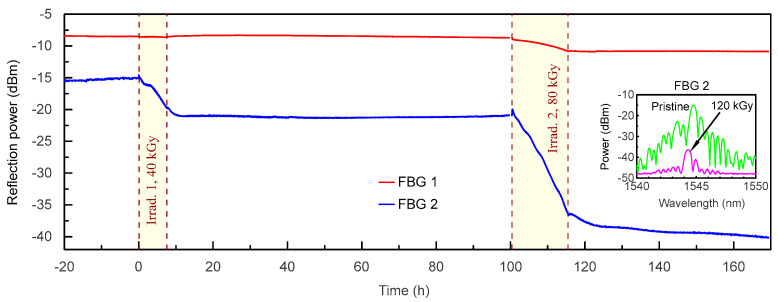
The Bragg wavelength reflection power evolution during the first (40 kGy) and the second (80 kGy) irradiation sessions.

First of all, let us compare the evolution of reflection peak power of FBGs 1 and 2 during the first two irradiation sessions (Figure 3). Due to a very short POF segment (3 cm) between the FBG 1 and the silica pigtail, the RIA in the connecting fiber had almost no effect during the first irradiation of 40 kGy, and there was quite a weak change of power (<2.5 dB) during the second irradiation of 80 kGy. In contrast, the reflection power of FBG 2 was strongly affected by the RIA in the connecting fiber: it decreased by 5 dB during the first irradiation and by 16 dB during the second irradiation.

The inset in Figure 3 shows the reflection spectrum of FBG 2 before the experiment and after the second irradiation (the spectra of the FBG 1 were shown in Figure 2a). This is the result of significantly longer POF between FBG 2 and the silica pigtail (1 m) compared to FBG 1. Indeed, according to [19,27], the radiation induces attenuation in PF-POF. Therefore, the RIA is stronger when using longer optical fiber samples. Thus, the higher irradiation dose, the shorter POF is preferable. It should be mentioned that the RIA during the second irradiation (−0.20 dB/kGy) was approximately two times higher than during the first irradiation (−0.11 dB/kGy) (the sensitivity was calculated using linear approximation of corresponding dependences). Additionally, we note that for both irradiation sessions, the reflection power continued to decrease shortly after the end of irradiation. However, after the first irradiation, the power stabilized and then even slightly recovered, while after the second irradiation, the power continued to slowly decrease during the whole measurement time.

**Figure 4:**
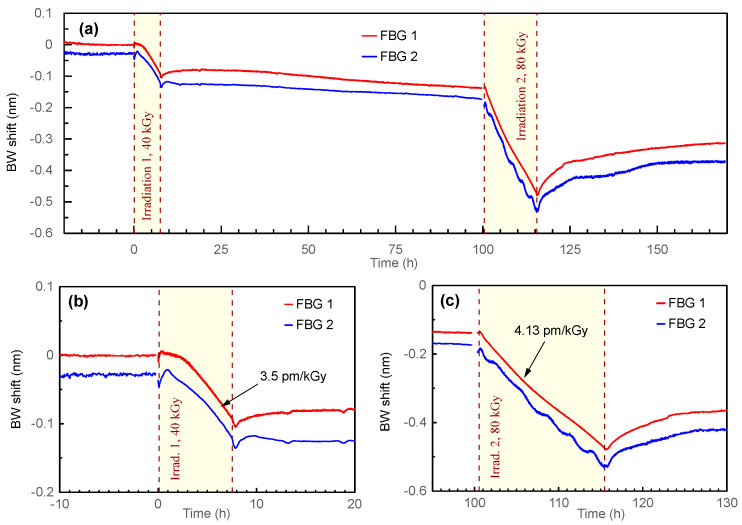
The Bragg wavelength evolution during the first and the second irradiation sessions: (**a**) general view; (**b**) irradiation 1 (40 kGy); (**c**) irradiation 2 (80 kGy). We introduced an additional BW shift of −30 pm for the FBG 2 for more convenient analysis of the graph.

Figure 4a shows the BW evolution of FBGs 1 and 2 during the first and the second irradiation sessions. We plotted the Bragg wavelength shift instead of the BW for more convenient analysis and comparison of the results. We also introduced a BW offset of −30 pm for FBG 2 to show the behavior of each grating independently. In general, both FBGs demonstrated a similar behavior. The reaction of the FBGs to the first irradiation (40 kGy) was the BW blue shift of 95–110 pm. After the irradiation, a weak recovery of the BW (≈20% of the irradiation BW shift) was seen during 10 h, and then a slow blue shift at a rate of ≈0.7 pm/h was observed. It should be noted that during the recovery process, the FBGs did not completely stabilize, even after 90 h after the first irradiation: the BW continued a slow decrease. The second irradiation (80 kGy) again caused the blue shift of the BW, however, its recovery was significantly stronger than after the first irradiation.

Figure 4b shows the data of the first irradiation in more detail. A slight BW rise (≈10 pm during 20 min for FBG 1 and ≈30 pm during 50 min for FBG 2) was seen after the start of irradiation. Then, the BW turned to a slow decrease over another 1.5–2 h, and finally, it reached the linearly decreasing part of the graph. The total BW shift during irradiation was −95 pm for FBG 1 and −110 pm for FBG 2. Both FBGs demonstrated the same sensitivity of −3.5 pm/kGy at the linear part. It should be mentioned that the BW of both FBGs experienced another 10-pm decrease immediately after irradiation. After that, the BW recovered as described in the previous paragraph.

The FBG evolution during the second irradiation (Figure 4c) demonstrated a weaker initial BW rise of ≈5 pm. Then, the BW of the FBG 1 decreased linearly with the slope of −4.13 pm/kGy. FBG 2 decreased with the same slope, however, it demonstrated noticeable parasitic fluctuations. The total BW change during the second irradiation was ≈−340 pm for both FBGs. The recovery BW shift was 130 pm at the end of the experiment (53 h after the end of irradiation 2) (i.e., 38% of the second irradiation BW shift).

**Figure 5:**
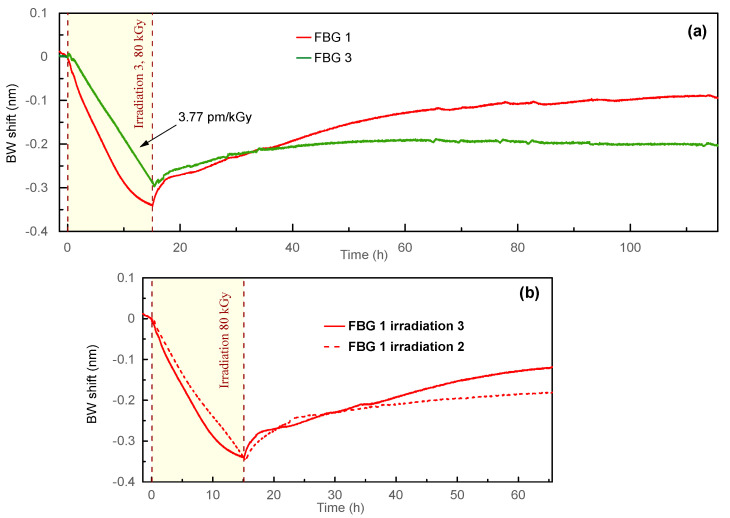
(**a**) The BW evolution during the third irradiation session (80 kGy); (**b**) comparison of irradiation sessions 2 and 3 (both 80 kGy) of FBG 1 (time offset was applied to the graph of irradiation 2 presented in Figure 4 to make it start at 0 h).

The general view of the third irradiation (80 kGy) is presented in Figure 5a. As a reminder, FBG 1 previously received a 120 kGy dose during the first (40 kGy) and the second (80 kGy) irradiation sessions, while FBG 3 was not irradiated yet (Table 1). Unlike FBGs 1 and 2, FBG 3 demonstrated a linear BW decrease with a sensitivity of −3.77 pm/kGy and a total BW shift of −300 pm. After irradiation, the BW continued to decrease for several minutes and then recovered. The total recovery BW shift of FBG 3 was 100 pm, that is, 33% of the total BW shift during irradiation; the recovery process saturated ≈40 h after irradiation.

The BW of FBG 1 decreased faster, with sensitivity at the linear part −5.45 pm/kGy. However, by the end of irradiation, the decrease slowed down, so the BW tended to reach some saturation value. The overall BW shift of the FBG 1 was −330 pm, that is, stronger than the case of FBG 3. The recovery BW shift of FBG 1 was also stronger: 250 pm, or 76% of the total BW shift during irradiation. The recovery process saturated approximately 100 h after irradiation.

Figure 5b compares irradiation sessions 2 and 3 (both 80 kGy) for FBG 1. It is seen that despite the nonlinear behavior of the BW during the third irradiation, the total BW shift was the same for irradiation sessions 2 and 3. However, the recovery was noticeably stronger for the third irradiation (76% versus 38% for the irradiation 2).

**Figure 6:**
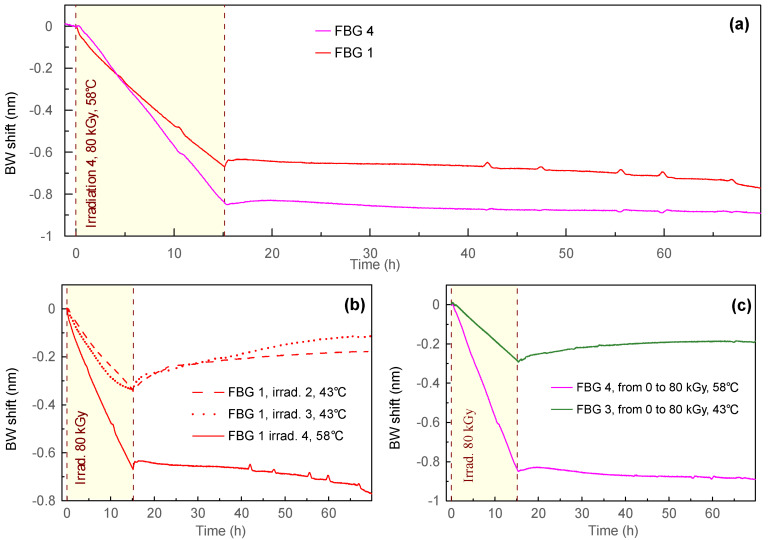
(**a**) The BW evolution during the fourth irradiation session (80 kGy, 58 °C); (**b**) comparison between irradiations 2, 3 and 4 for FBG 1 (time offset was applied to the graph of irradiation 2 to make it start at 0 h); (**c**) comparison of irradiations 3 (FBG 3) and 4 (FBG 4) at 43 °C and 58 °C correspondingly.

The BW evolution during the fourth irradiation session (80 kGy, 58 °C) is presented in Figure 6a. FBG 1 previously received the total dose of 200 kGy during irradiation sessions 1–3, while FBG 4 was not irradiated before the experiment. Both FBGs showed a linear response to the received doses. The sensitivity calculated using linear approximation of the BW dependency on received dose was −7.87 pm/kGy for FBG 1 and −10.6 pm/kGy for FBG 4. This was a significantly stronger value than that obtained at 43 °C.

Figure 6b shows a comparison of the BW dependences of FBG 1 during irradiations 2–4 (all with the doses of 80 kGy). The total BW shift of the FBG 1 for the 4th irradiation is −670 pm that is approximately 2 times stronger than for irradiation sessions 2 and 3. The recovery process after the fourth irradiation principally differed compared to irradiation sessions 2 and 3: after a slight recovery right after irradiation 4, the BW decreased with time, while a strong positive recovery was observed after irradiation sessions 2 and 3.

Figure 6c shows the comparison of the BW dependences of FBGs 3 and 4 during irradiation up to 80 kGy at temperatures of 43 °C and 58 °C correspondingly (both FBGs were not irradiated before the corresponding experiment). The total BW shift during irradiation for FBG 4 was −850 pm, which was 2.8 times higher than for FBG 3. It should be mentioned that the recovery behavior was again different for different temperatures. At 58 °C, a very small recovery directly after irradiation was seen, however, it changed to a slow decrease in the BW during the rest of the experiment, while positive recovery was observed during the third irradiation session at 43 °C.

## 4. Discussion

The majority of experiments demonstrated a linear dependency of the BW on the received dose. However, the cases of deviation from this behavior should be discussed. At the beginning of the first irradiation session, both FBGs 1 and 2 first showed a certain rise in the BW. Only then, the BW did start to decrease, finally reaching linear dependency on the received dose. The reason of this behavior could be the temperature stabilization after the start of irradiation. As stated in Section 2, before the first irradiation, we stabilized the temperature at 40 °C. During the first irradiation, the temperature rose by ≈3 °C. To minimize this temperature change, we increased the temperature of the oven up to 42 °C, so the temperature change during further irradiation sessions decreased down to ≈1 °C. Thus, higher amplitude of temperature stabilization during the first irradiation could cause the nonlinear BW response at the beginning of the experiment, while linear dependences in further experiments were achieved by better temperature stabilization (ΔT ≈ 1 °C during irradiation).

Another case of a deviation from the linear response was FBG 1 during irradiation 3. By the end of the irradiation, a tendency of the BW to saturate was observed. The fiber previously received a 120 kGy dose, and the saturation can be linked to a limited capacity of possible defects in the material. However, the next irradiation of FBG 1 at an increased temperature of 58 °C again demonstrated a linear response. It can be explained by more efficient recovery processes during stabilization at increased temperature, which leads to the rise in the number of potential defects that are possible to create during irradiation. Alternatively, a higher temperature itself could provide a higher number of potential defects that are possible to create independently of the previous recovery process.

The results of FBG 1, which was irradiated during all irradiation sessions, showed that the sensitivity at the linear parts of the graphs slightly changed with the number of irradiation sessions (−3.5 pm/kGy, −4.13 pm/kGy, and −5.45 pm/kGy for irradiations 1, 2, and 3, respectively). The lower value of sensitivity in the first irradiation can be explained by the reaction to temperature stabilization, as discussed above. The third irradiation provides a total dose, which almost led to the BW saturation. Therefore, doses exceeding 120 kGy turn the response to nonlinear at temperatures around 40 °C. Thus, the sensitivity value of −4.13 pm/kGy obtained from the second irradiation session seems the most reliable: the temperature change during irradiation was ≈1 °C, which was three times smaller than during irradiation 1, the total dose was far enough from the BW nonlinearity effect, and both FBGs 1 and 2 demonstrated the same sensitivity. Moreover, the hermetic container was closed and stabilized under water during more than 100 h, since the FBGs were not changed between the first and second irradiation. Additionally, the sensitivity of −3.77 pm/kGy obtained for FBG 3 also seems reliable, since the temperature was well-stabilized, and the response was linear. Hence, we used the mean sensitivity of −3.95 pm/kGy calculated by averaging the values for FBGs 1 and 2 (irradiation 2) and FBG 3 (irradiation 3) as a representative value. Nevertheless, one continuous irradiation session with strong dose, for example, 200–300 kGy, would clarify the obtained results.

For precise calibration of the sensor, the sensitivity to radiation should be measured at well-stabilized temperatures. The sensitivity should be reproducible for different CYTOP FBGs, provided that the temperature and the dose rate are the same for different FBG samples. Therefore, pristine FBGs could be used for sensing without pre-calibration of each grating (i.e., using earlier obtained sensitivities to temperature and radiation).

Possible physical mechanisms that can lead to the BW blue shift are fiber shrinkage under irradiation, which reduces the FBG pitch, and the decrease of the core refractive index. It is not obvious which of these two mechanisms are involved during irradiation. In the case of length shortening, it can occur with the CYTOP core/cladding structure or (and) with the XYLEX overclad as well (separately or together). Therefore, the measurement of fiber length change before and after irradiation (even if it shows some decrease in the length), could not answer exactly the origin of the fiber length suppression: suppression of CYTOP core/cladding structure, suppression of XYLEX overclad, or both.

The BW shift under irradiation could also be related to the same mechanisms inducing the radiation induced attenuation. Stajanka et al. [19] suggest that two primary mechanisms are involved: (1) absorption on unpaired electrons of free radicals, and (2) the formation of conjugated systems. The first process is thermally unstable, so the transmission recovery takes place after irradiation. The second process is more stable and causes permanent losses. Therefore, we can suppose that the presence of these two mechanisms can be a reason for the different post-irradiation behavior of the FBGs: almost no recovery of the BW after the first irradiation (permanent fiber changes) and subsequent BW recovery after the next irradiation sessions (unstable fiber changes). Additionally, increased temperature eliminated the post-irradiation recovery stage (Figure 6a), which indicates a permanent type of fiber changes.

Previously, FBGs inscribed in a standard multimode CYTOP fiber with a core diameter of 120 µm and polycarbonate overclad with a diameter of 490 µm were investigated for gamma radiation response [30]. The authors obtained significantly higher sensitivity of −29.9 pm/kGy at a dose rate of 635 Gy/h, which was more than eight times less than the dose rate used in this work. Additionally, Olusoji et al. [28] reported the sensitivity of ≈−7 pm/Gy for the FBG inscribed in a standard CYTOP fiber with a core diameter of 50 µm under low-dose X-ray irradiation with a dose rate of 106 Gy/h and a received dose of 9 Gy. The results were very different, and therefore, the reasons for such differences must be discussed. The reasons could be the difference in the dose rate, the type of radiation (X-ray or gamma), the temperature during irradiation, the diameters of the core, the cladding and the overclad of the fibers, the type of the overclad material, and the history of the fiber’s climatic conditions before irradiation. The latter includes, in particular, the temperature annealing: the fiber exposure at the increased temperature led to a permanent blue shift of the BW, if preliminary annealing was not performed. Therefore, since the temperature increases during irradiation, the reason for the BW blue shift of the not annealed fiber can not only be the radiation itself, but also the temperature.

It is also important to compare the gamma radiation effect of CYTOP FBGs investigated in the present work with FBGs inscribed in silica fibers. Unlike CYTOP FBGs, silica FBGs demonstrate positive BW shift under irradiation [10,32,33]. They also demonstrate a nonlinear response to the obtained dose: the sensitivity decreased with dose, so the BW tended to saturate at certain doses [10,32]. Depending on the fiber type, some FBGs demonstrated low sensitivity (15–25 pm BW shift at 100 kGy received dose) and fast saturation (at 20–30 kGy). They are, therefore, more appropriate for radiation-resistant sensing purposes in radiation environments. Another group of FBGs demonstrates higher sensitivity to gamma radiation and saturation at higher doses, which is more suitable for gamma radiation sensing purposes. According to review [10], these high sensitive FBGs demonstrate the BW shift up to 200 pm as a response to the received dose of 100 kGy. The CYTOP FBGs demonstrated a BW shift of about −300 pm at the 80 kGy received dose, which was higher than the BW shift of silica FBGs. Moreover, the obtained results show the linear response to received dose, which is attractive for sensing compared to silica counterparts. At the same time as the silica FBG case, the sensitivity of the CYTOP FBGs was temperature dependent. Therefore, temperature dependence of the sensitivity should be compensated for practical applications. The same advantage of linearity is true for CYTOP FBGs compared to silica LPGs. However, the sensitivity of some types of LPGs was much stronger (10-nm dip shift at 100 kGy dose for chiral LPGs and 80-nm dip shift at a 65 kGy dose for turn-around-point LPGs) [10,12,13,14]. The necessity of the transmission signal monitoring in the case of LPGs can bring additional inconveniences compared to the reflection type of interrogation with FBGs. The sensor based on a metallo-dielectric resonator located at the optical fiber facet (lab-on-fiber concept) demonstrated the sensing ability in a very wide range of doses (MGy range) with, however, a lower sensitivity (−0.6 pm/kGy versus −3.95 pm/kGy for CYTOP FBGs) [20].

The graph of reflection peak power (Figure 3) indicates that the length of the POF between the FBG and silica pigtail should be carefully selected. On one hand, the reflection peak should have enough power before and after irradiation for correct interrogation. Ideally, the POF length between the FBG and the silica pigtail should be minimized, as was conducted for example, in the FBG 1 sample. On the other hand, the radiation induced attenuation could be used as an additional sensing mechanism along with the BW shift: the case of FBG 2 having a 1-m POF segment showed the BW peak attenuation as a result of irradiation. Therefore, the dose received by the entire POF can be estimated, so the POF could serve as a distributed sensing element in addition to FBG as a point sensor. It should be mentioned that the evolution of the reflection power of FBG 2 during the irradiation sessions showed deviations from the linear dependency. Additionally, the reflection power behavior during the relaxation process after both irradiation sessions was different: after the first irradiation of 40 kGy, where the reflection power stabilized, while after the second irradiation, it continued to decrease. Nevertheless, we suppose that the most important is linearity and stability of the response during irradiation. It can be improved by choosing ideally inscribed FBGs (well centered, without tilts and displacements) and by paying additional attention to the launching conditions to provide exclusive fundamental mode launch and to avoid significant power in higher order modes. Additionally, the length of the POF should be limited to reduce the effect of mode coupling and power redistribution to higher order modes. The last condition requires a compromise between high sensitivity (due to longer fiber) and high quality of the FBG response (due to shorter fiber).

## 5. Conclusions

In this work, we investigated the response of FBGs inscribed in a few-mode CYTOP fiber to gamma radiation. The FBGs demonstrate a linear response to the received dose with a mean sensitivity of −3.95 pm/kGy at a dose rate of 5.3 kGy/h and temperature of 43 °C. The linearity was maintained up to 120 kGy dose, and then the BW tended to saturate. Temperature strongly improved the sensitivity: it rose 2.8 times (for the previously not irradiated FBG sample) when the temperature was increased from 43 °C to 58 °C. Increased temperature also broadens the range of doses with linear response: the FBG, which previously received a 200 kGy total dose at 43 °C and demonstrated a nonlinear response after receiving a 120 kGy dose, showed a linear response again at 58 °C for another 80-kGy dose. The magnitude of the BW recovery increased with the received dose, however, it was weakened with temperature. The length of the irradiated POF (between the SMF and the FBG) affects the reflection peak’s amplitude with the received dose due to the radiation-induced attenuation. The peak’s power, therefore, can be used as an additional indicator of the received by the entire POF dose.

To conclude, FBGs inscribed in CYTOP fibers are attractive for dosimetry tasks thanks to their linear response in a broad dose range and respectively strong sensitivity. However, they demonstrate significant temperature dependency of the sensitivity. Future work should be focused on the influence of the dose rate on the sensing characteristics, on the influence of annealing, and on the influence of irradiation interruptions on the range of doses providing a linear response.

## Figures and Tables

**Table 1:** Order of the irradiation sessions with corresponding doses and FBGs.

Irradiation Session (Dose)	BW (nm)	1 (40 kGy)	2 (80 kGy)	3 (80 kGy)	4 (80 kGy, 58 °C) ^1^	Total Dose (kGy)
FBG 1	1552.5	√	√	√	√	280
FBG 2	1545.3	√	√	-	-	120
FBG 3	1543.2	-	-	√	-	80
FBG 4	1551.0	-	-	-	√	80

^1^ Irradiation 1 was conducted at 40 °C, irradiations 2 and 3 were conducted at 42 °C.

## Data Availability

Not applicable.
